# Supplementary material for: Redox-Regulation of α-Globin in Vascular Physiology
Source: Antioxidants (Basel). 2022 Jan 14;11(1):159. doi: 10.3390/antiox11010159 (PMC8773178; doi:10.3390/antiox11010159)
Supplement: Supplementary file 1 [file antioxidants-11-00159-s001.zip › antioxidants-1458714-supplementary.pdf]

Supplemental Table S1. Antibodies used in this study.

Antibody	Type	Concentration	Supplier, reference no.
α-Globin	Polyclonal	10 µg/mL (histology)	Abcam, Ab92492
α-Globin	Polyclonal	(Western blot)	Home-made
eNOS	Monoclonal	1 µg/mL (histology)<br>0.2 µg/mL (Western blot)	BD 610297
CyB5a	Polyclonal	4 µg/mL (histology)<br>0.4 µg/mL (Western blot)	Proteintech, 12365-1-AP
CyB5b	Polyclonal	2.5 µg/mL (histology)<br>0.25 µg/mL (Western blot)	Proteintech, 15469-1-AP
CyB5R3	Polyclonal	0.4 µg/mL (histology)<br>0.2 µg/mL (Western blot)	Proteintech, 10894-1-AP
Goat anti-rabbit IgG	HRP conjugate	0.05 µg/mL	Jackson ImmunoResearch Laboratories, 111-035-003
Goat anti-mouse IgG	HRP conjugate	0.05 µg/mL	Jackson ImmunoResearch Laboratories, 115-035-003

Supplemental Table S2. Oligonucleotide primers used for RT-qPCR.

GENE	ACCESSION	FORWARD PRIMER, 5′→ 3′	REVERSE PRIMER, 5′→ 3′
HBA	NM_008218	GCTCTCTGGGGAAGACAAAA	GCCGTGGCTTACATCAAAGT
HBB	NM_001278161	TGTTGACTCACAACCCCAGA	CTGGGTCCAAGGGTAGACAA
NOS3	NM_008713	GCACCCAGAGCTTTTCTTTG	GTCAACCGAACGAAGTGACA
CYB5A	NM_025797	CTGGGTGATCCTGCATCATA	ACAGTTCTCGTGCATCCGTA
CYB5B	NM_025558	CTGCGATGAGAGGAGGAGAC	CTCTGGCGGATGAAAGACTC
CYB5R3	NM_029787	GGAAGAGAGAGGGAGGGAGA	GGCAGGTAGGTGGTTAGGTG
CYGB	NM_030206	GAGTTGGGGTGAGTCAGCTT	CCGTGGAGACCAGGTAGATG
ACTB	NM_007393	CCATCTACGAGGGCTATGCT	TTTGATGTCACGCACGATTT

HBA, α-globin; *HBB*, β-globin; *NOS3*, eNOS; *CYB5A*, cytochrome b_5 type A; *CYB5B*, cytochrome b_5 type B; *CYB5R3*, cytochrome b_5 reductase 3; *CYGB*, cytoglobin; *ACTB*, β-actin

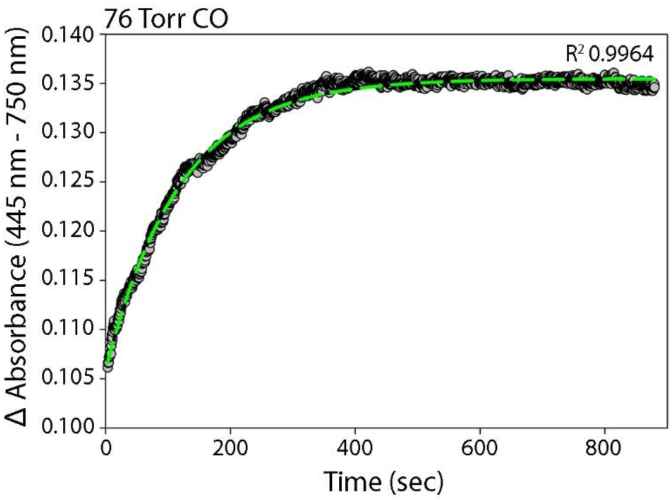

Supplemental Figure S1. eNOS reduction. eNOS reduction kinetics after mixing NADPH under 76 Torr of CO. The dashed lines represent the exponential rise fit, with the associated determination coefficient (R2).

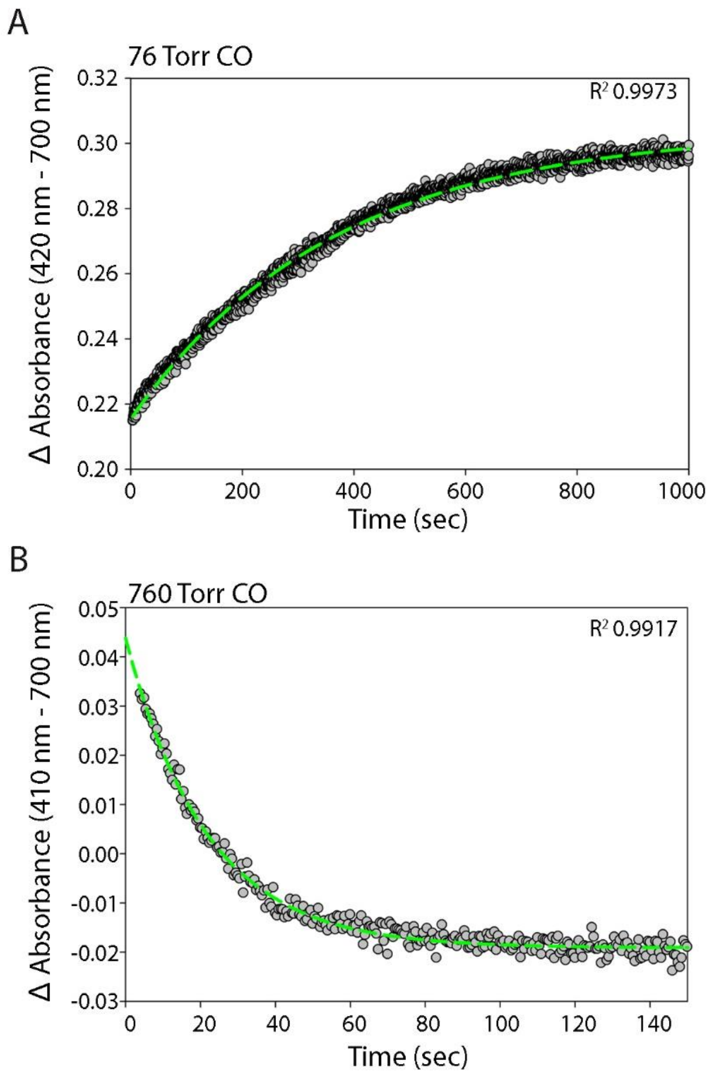

Supplemental Figure S2. α-Globin reduction. α-Globin–AHSP reduction kinetics, after mixing with CyB5R3/CyB5a (A) or eNOS (B) pre-incubated under anaerobic conditions in the presence of NADH or NADPH, respectively, under CO. The dashed lines represent the exponential rise or decay fits, with the associated determination coefficient (R2).
